# Supplementary material for: Coexistence of Retinitis Pigmentosa and Ataxia in Patients with PHARC, PCARP, and Oliver–McFarlane Syndromes
Source: Int J Mol Sci. 2024 May 25;25(11):5759. doi: 10.3390/ijms25115759 (PMC11172263; doi:10.3390/ijms25115759)
Supplement: Supplementary file 1 [file ijms-25-05759-s001.zip › ijms-2980415-supplementary.pdf]

Table S1 PCR and qPCR primers.

Primer	Sequence (5' to 3', Hg38)	Genomic coordinates	Index patient
**PCR, Sanger sequencing, c.1063C>T**			P1
ABHD12_F	AGCAGTGTATGTGGGACTCTG	chr20:25302155-25302467	
ABHD12_R	TGCACGTTAGGTGTGAGCCA		
**qPCR, deletion**			
PHARC_DEL_F1	ACATAAAAATCCACACCCTGTCC	chr20:24543516-24543597	
PHARC_DEL_R1	TGACAAACACATCCTGTGACCT		
PHARC_DEL_F2	CTGCAGTTCCGTGGTTATTGAT	chr20:25320427-25320516	
PHARC_DEL_R2	TGCAGACCCCATCTAACTACAG		
PHARC_DEL_F3	TGCAGACTTACCTGTGTCCCA	chr20:25441298- 25441377	
PHARC_DEL_R3	TAGATCTGCTCCAACCTGCTTC		
PHARC_DEL_F4	TGAACTTCGAAAGGAGGTCCG	chr20:25616231-25616315	
PHARC_DEL_R4	ACAGGCACAAGCCTCAATCT		
**PCR, Sanger sequencing, c.648C>A, c.733A>T**			P2
FLVCR1_F	GTCTTCGAGGGCTTCTACGG	chr1:212858852-212859335	
FLVCR1_R	GGCCTCCTATTCAACGGCTT		
**PCR, Sanger sequencing, c.1387C>T**			P3
PNPLA6_F1	TCCGACTTCGACATGGCCT	chr19:7542581-7543190	
PNPLA6_R1	TGGGAGAAGGTGCTGGTCTT		
**PCR, Sanger sequencing, c.3343G>A**			
PNPLA6_F2	TTAACACGAGTGACCGTCCA	chr19:7556817-7557473	
PNPLA6_R2	CTGTCCACAGGCACATTCAT		
**Q-PCR oligonucleotide primers used to establish breakpoints of the CNVs**			P1
PHARC_DEL_F5	TCTCCCCCAAAGTGCTAAGA	chr20:25317196-25317277	
PHARC_DEL_R5	CTTGGGTCCCCCTAAGTAGC		
PHARC_DEL_F6	AGTTCTGCAGTGCCCATCAC	chr20:25318626-25318705	
PHARC_DEL_R6	ACGGCATCCCTTTCTAACCT		
PHARC_DEL_F7	ATGGCTTGTCCTTTCCACTG	chr20:25319243-25319322	

PHARC_DEL_R7	GCAATCACATGGTGTGGAAG		
PHARC_DEL_F8	CTCCGAGGAGGGGTCATAGT	chr20:25319635-25319721	
PHARC_DEL_R8	AAAACAAGCTGCCAGTGAGC		
PHARC_DEL_F9	TCATCTTTGCTGTGGAGCAT	chr20:25320100-25320180	
PHARC_DEL_R9	AGGTTGCTCCTGCTGGTTT		
PHARC_DEL_F10	GGATGAGGACAGAAGGGACA	chr20:25320285-25320373	
PHARC_DEL_R10	GGCGTTCTTCCACCAGACT		
PHARC_DEL_F11	AGCTGCAGACCTGGGATTCT	chr20:25504994-25505075	
PHARC_DEL_R11	ATCTGGCTCTCTGGGGTGT		
PHARC_DEL_F12	GGCAGAACGTCCTTCTTTCT	chr20:25510404-25510484	
PHARC_DEL_R12	GAAGCAAGGGAGGAGTCACA		
PHARC_DEL_F13	CTGCTGAAGCAGAACTGCAA	chr20:25507532-25507615	
PHARC_DEL_R13	CTCATCCACACCACCTAGCC		
PHARC_DEL_F14	GCGATTTCAGATACCCCAGA	chr20:25506758-25506846	
PHARC_DEL_R14	TGCATATTGACCTCACACCA		
PHARC_DEL_F15	TTTTCCCCATTACAGGCAGA	chr20:25505841-25505923	
PHARC_DEL_R15	TGCCACAGTTGTTTGCACTT		
PHARC_DEL_F16	GTCACTCGGTCCTCATCCTC	chr20:25505081-25505162	
PHARC_DEL_R16	AAGTGTGACGAACCACGATG		
**Sequencing oligonucleotide primers used to establish breakpoints of the CNVs**			P1
PHARC_DEL_F17	AAGTGCAAACAACTGTGGCA	chr20:25318689-25505860	
PHARC_DEL_R17	ATGGGCACTGCAGAACTGAA		
PHARC_DEL_F18	AACTGTAGTCACCGTGCTGT	chr20:25318689-25505410	
PHARC_DEL_R18	ATGGGCACTGCAGAACTGAA		
PHARC_DEL_F19	CCCCTTTCCTTAGATCCCACA	chr20:25318689-25505273	
PHARC_DEL_R19	ATGGGCACTGCAGAACTGAA		
**qPCR - reference genes**			P1
ALB_F	TGAAATGGCTGACTGCTGTG	chr4:73408650-73408732	
ALB_R	GGAGGTTTGGGTTGTCATCT		
F8_F	TTTCCATTCAACACCTCAGTCGT	chrX:154999491-154999575	

F8_R	GCCTTGGCTTAGCGATGTTG		

Supplementary material

P1

Charcot-Marie-Tooth Neuropathy Panel Plus, 86 genes

Genes: AARS, AIFM1, AMACR, ARHGEF10, ATL1, ATL3, ATP7A, BAG3, BSCL2, C12ORF65, CCT5, COX10, COX6A1, CTDP1, DCAF8, DCTN1, DHTKD1, DNM2, DNMT1, DST, DYNC1H1, EGR2, FAM134B, FBLN5, FGD4, FIG4, FXN, GAN, GARS, GDAP1, GJB1, GNB4, GNE, HADHB, HARS, HINT1, HK1, HSPB1, HSPB8, IGHMBP2, INF2, KARS, KIF1A, KIF1B, KIF5A, LDB3, LITAF, LMNA, LRSAM1, MARS, MED25, MFN2, MPZ, MTMR2, MYOT, NDRG1, NEFL, NGF, NTRK1, PDK3, PLEKHG5, PMP22, POLG, PRPS1, PRX, RAB7A, REEP1, SAC5, S8F1, SBF2, SCN9A, SETX, SH3TC2, SLC12A6, SMAD3, SPG11, SPTLC1, SPTLC2, SURF1, TFG, TRIM2, TRPV4, TYMP, VCP, WNK1, YARS.

P3

Retinal Panel NGS, 317 genes

ABCA4, ABCC6(*), ABHD12, ACBD5, ACO2, ADAM9, ADAMTS18, ADGRA3, ADGRV1, ADIPOR1(*), AFG3L2(*), AGBL5, AHI1, AHR, AIPL1, ALMS1(*), ARHGEF18, ARL13B, ARL2BP, ARL3, ARL6, ARMC9, ARMS2, ARSG, ASRGL1, ATF6, ATXN7, B9D1, B9D2, BBIP1, BBS1, BBS10, BBS12, BBS2, BBS4, BBS5, BBS7, BBS9, BEST1, C12orf65, C1QTNF5, C2(*), C2CD3, C3, C8orf37, CA4, CABP4, CACNA1F, CACNA2D4, CAPN5, CC2D2A, CCDC28B, CCT2, CDH23, CDH3, CDHR1, CELSR2, CEP104, CEP120, CEP164, CEP19, CEP250, CEP290(*), CEP41, CEP78, CERKL, CFAP410, CFB, CFH(*), CHM, CIB2, CLCC1, CLN3, CLRN1, CLUAP1, CNGA1, CNGA3, CNGB1, CNGB3, CNNM4, COL11A1, COL2A1, COL9A1, CPLANE1, CRB1, CRX, CSPP1, CTNNA1, CWC27, CYP4V2, DHDDS, DHX38, DMD, DRAM2, DTHD1, DYNC2H1, EFEMP1, ELOVL1, ELOVL4, EMC1, ENSA, ERCC6(*), ESPN(*), EXOC8, EXOSC2, EYS(*), FAM149B1, FAM161A, FBLN5, FLVCR1, FRMD7, FSCN2, FZD4, GDF6, GNAS, GNAT1, GNAT2, GNB3, GNPTG, GPR143, GPR179, GRK1, GRM6, GUCA1A, GUCA1B, GUCY2D, HARS1, HGSNAT, HK1, HMCN1, HMX1, HTRA1, HYLS1, IDH3B, IFT140, IFT172, IFT27, IFT74, IFT81, IMPDH1, IMPG1, IMPG2, INPP5E, INVS, IQCB1, ITM2B, JAG1, KCNJ13, KCNV2, KIAA0556, KIAA0586, KIAA0753, KIAA1549, KIF11, KIF3B, KIF7, KIZ, KLHL7, LAMA1, LCA5, LRAT, LRIT3, LRP5(*), LZTFL1, MAK, MAPKAPK3, MERTK, MFN2, MFRP, MFSD8, MIEF1, MKKS, MKS1, MTTP, MVK, MYO7A, NBAS, NDP, NEK2, NEUROD1, NMNAT1, NPHP1, NPHP3, NPHP4, NR2E3, NR2F1, NRL, NYX, OAT, OFD1, OPA1, OPA3, OPN1LW, OPN1MW, OPN1SW, OTX2, PANK2, PAX2, PCARE, PCDH15, PCYT1A, PDE6A, PDE6B, PDE6C, PDE6D, PDE6G, PDE6H, PDZD7, PEX1, PEX2, PEX7, PGK1, PHF6, PHYH, PIBF1, PITPNM3, PLA2G5, PLK4, PNPLA6, POC1B, POC5, POMGNT1, PPT1, PRCD, PRDM13, PROM1, PROS1(*), PRPF3, PRPF31, PRPF4, PRPF6, PRPF8, PRPH2, PRPS1(*), RAB28, RAX2, RB1, RBP3, RBP4, RCBTB1, RD3, RDH11, RDH12, RDH5, REEP6, RGR, RGS9, RGS9BP, RHO, RIMS1, RLBP1, ROM1, RP1, RP1L1, RP2, RP9, RPE65, RPGR, RPGRIP1, RPGRIP1L, RS1, RTN4IP1, SAG, SAMD11, SDCCAG8, SEMA4A, SLC24A1, SLC25A46, SLC4A7, SLC7A14, SNRNP200, SPATA7, SPP2, SUFU, TCTN1, TCTN2, TCTN3, TEAD1, TIMM8A(*), TIMP3, TLR3, TLR4, TMEM107, TMEM126A, TMEM138, TMEM216, TMEM231, TMEM237, TMEM67, TOPORS, TRAF3IP1, TREX1, TRIM32, TRNT1, TRPM1, TSPAN12, TTC21B, TTC8, TTLL5, TTPA, TUB, TUBGCP4, TUBGCP6, TULP1, UNC119, USH1C, USH1G, USH2A, VCAN, WDPCP, WDR19, WDR34, WFS1,WHRN, ZNF408, ZNF423, ZNF513.

Figure S1. Breakpoints identification

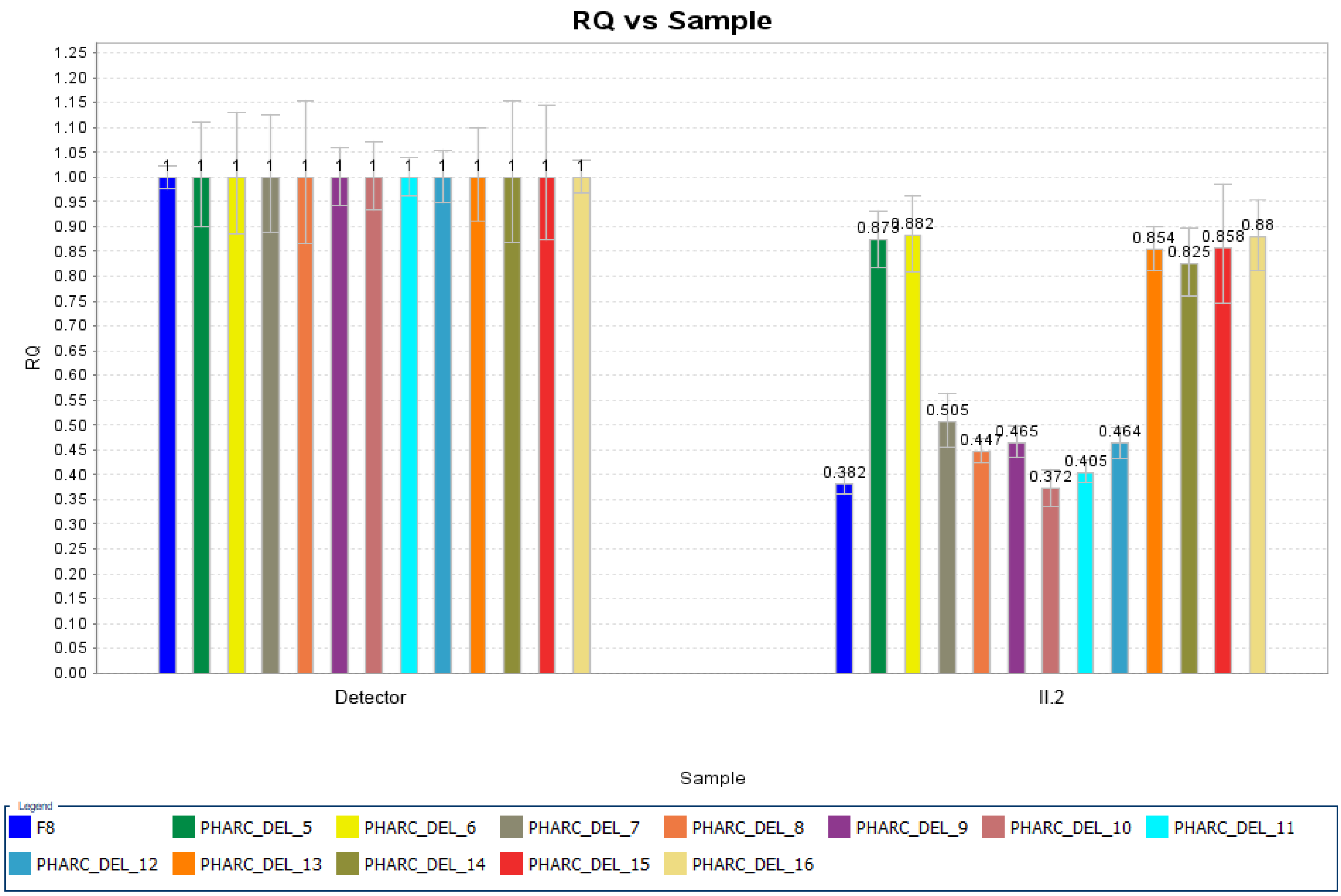

In order to identify the breakpoints, a series of qPCR reactions were performed using 12 pairs of primers, different from those previously used to confirm the deletion. Conducting this experiment enabled the approximation of the sought-after break site.

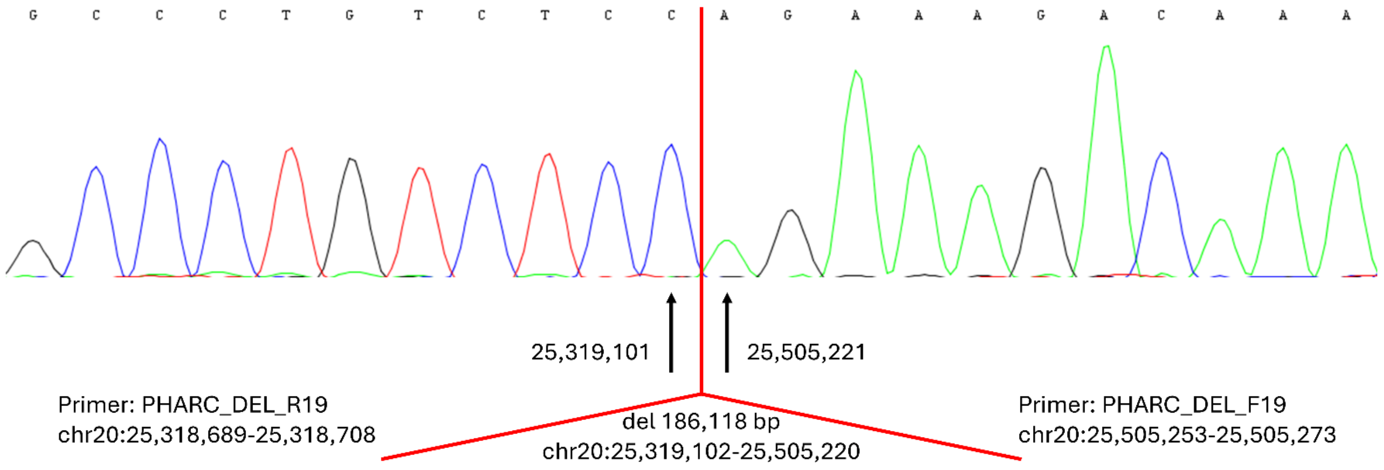

Figure S2. For precise determination of breakpoints new PCR primers were designed, and then the obtained PCR product was sequenced using the Sanger method. Chromatogram showing breakpoints of the deletions identified in family 1. Reference sequences were obtained from the UCSC Genome Browser on Human (GRCh38/hg38) Assembly.

Table S2 Genomic targets of qPCR primers used to confirm deletion and to identify breakpoints.

**Primer**	**Sequence (5’ to 3’, Hg38)**	**Targeted sequence**
PHARC_DEL_F1	ACATAAAAATCCACACCCTGTCC	*SYNDIG1* exon2
PHARC_DEL_R1	TGACAAACACATCCTGTGACCT	*SYNDIG1* intron 2-3
PHARC_DEL_F2	CTGCAGTTCCGTGGTTATTGAT	*ABHD12* Intron 3-4
PHARC_DEL_R2	TGCAGACCCCATCTAACTACAG	*ABHD12* intron 3-4
PHARC_DEL_F3	TGCAGACTTACCTGTGTCCCA	*GINS1* intron 5-6
PHARC_DEL_R3	TAGATCTGCTCCAACCTGCTTC	*GINS1* intron 5-6
PHARC_DEL_F4	TGAACTTCGAAAGGAGGTCCG	*NANP* exon 2
PHARC_DEL_R4	ACAGGCACAAGCCTCAATCT	*NANP* exon2
PHARC_DEL_F5	TCTCCCCCAAAGTGCTAAGA	*ABHD12* intron 4-5
PHARC_DEL_R5	CTTGGGTCCCCCTAAGTAGC	*ABHD12* intron 4-5
PHARC_DEL_F6	AGTTCTGCAGTGCCCATCAC	*ABHD12* intron 4-5
PHARC_DEL_R6	ACGGCATCCCTTTCTAACCT	*ABHD12* intron 4-5
PHARC_DEL_F7	ATGGCTTGTCCTTTCCACTG	*ABHD12* intron 4-5
PHARC_DEL_R7	GCAATCACATGGTGTGGAAG	*ABHD12* intron 4-5
PHARC_DEL_F8	CTCCGAGGAGGGGTCATAGT	*ABHD12* intron 4-5
PHARC_DEL_R8	AAAACAAGCTGCCAGTGAGC	*ABHD12* intron 4-5
PHARC_DEL_F9	TCATCTTTGCTGTGGAGCAT	*ABHD12* intron 4-5
PHARC_DEL_R9	AGGTTGCTCCTGCTGGTTT	*ABHD12* intron 4-5
PHARC_DEL_F10	GGATGAGGACAGAAGGGACA	*ABHD12* intron 3-4
PHARC_DEL_R10	GGCGTTCTTCCACCAGACT	*ABHD12* exon 4
PHARC_DEL_F11	AGCTGCAGACCTGGGATTCT	*NINL* exon 6
PHARC_DEL_R11	ATCTGGCTCTCTGGGGTGT	*NINL* exon 6
PHARC_DEL_F12	GGCAGAACGTCCTTCTTTCT	*NINL* intron 5-6
PHARC_DEL_R12	GAAGCAAGGGAGGAGTCACA	*NINL* intron 5-6
PHARC_DEL_F13	CTGCTGAAGCAGAACTGCAA	*NINL* intron 5-6
PHARC_DEL_R13	CTCATCCACACCACCTAGCC	*NINL* intron 5-6

PHARC_DEL_F14	GCGATTTCAGATACCCCAGA	*NINL* intron 5-6
PHARC_DEL_R14	TGCATATTGACCTCACACCA	*NINL* intron 5-6
PHARC_DEL_F15	TTTTCCCCATTACAGGCAGA	*NINL* intron 5-6
PHARC_DEL_R15	TGCCACAGTTGTTTGCACTT	*NINL* intron 5-6
PHARC_DEL_F16	GTCACTCGGTCCTCATCCTC	*NINL* intron 5-6
PHARC_DEL_R16	AAGTGTGACGAACCACGATG	*NINL* intron 5-6

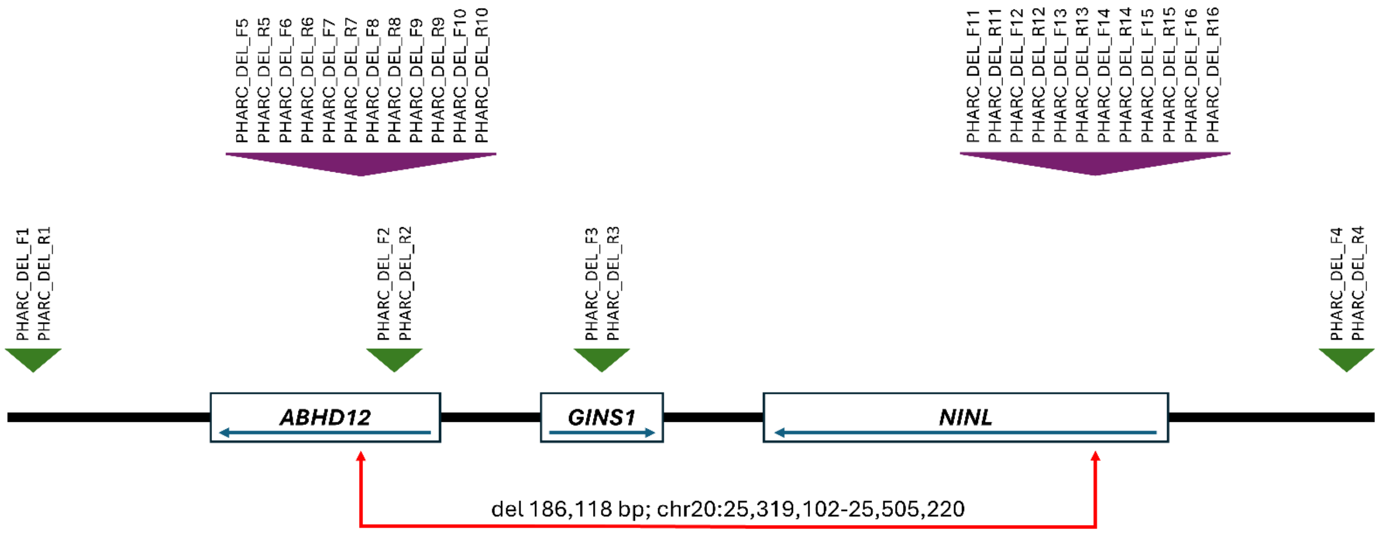

Figure S3. Schematic representation of the location of qPCR primers used. The green triangle shows the primers used to confirm the deletion, while the purple one shows those used to narrow down the sequence with the breakpoints.

A.

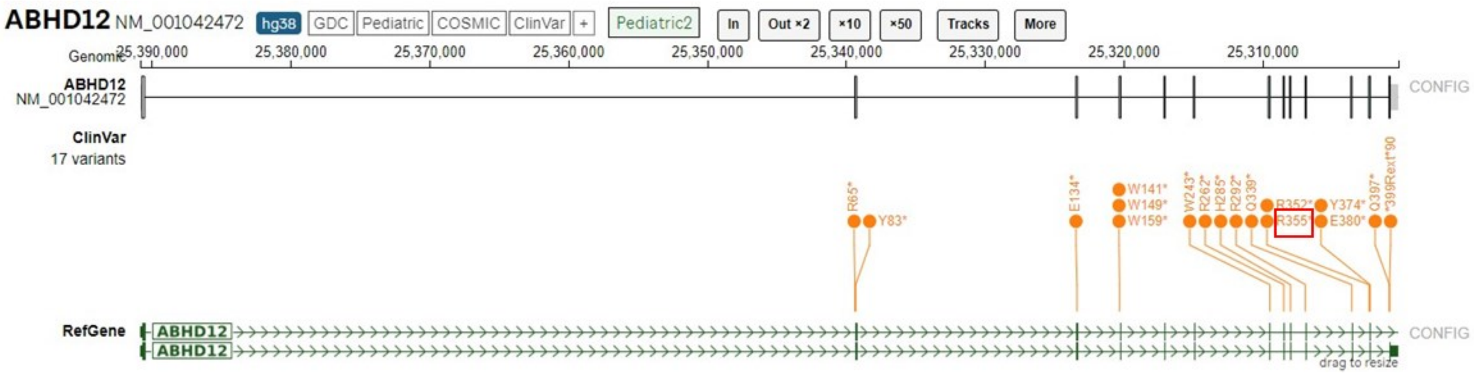

B.

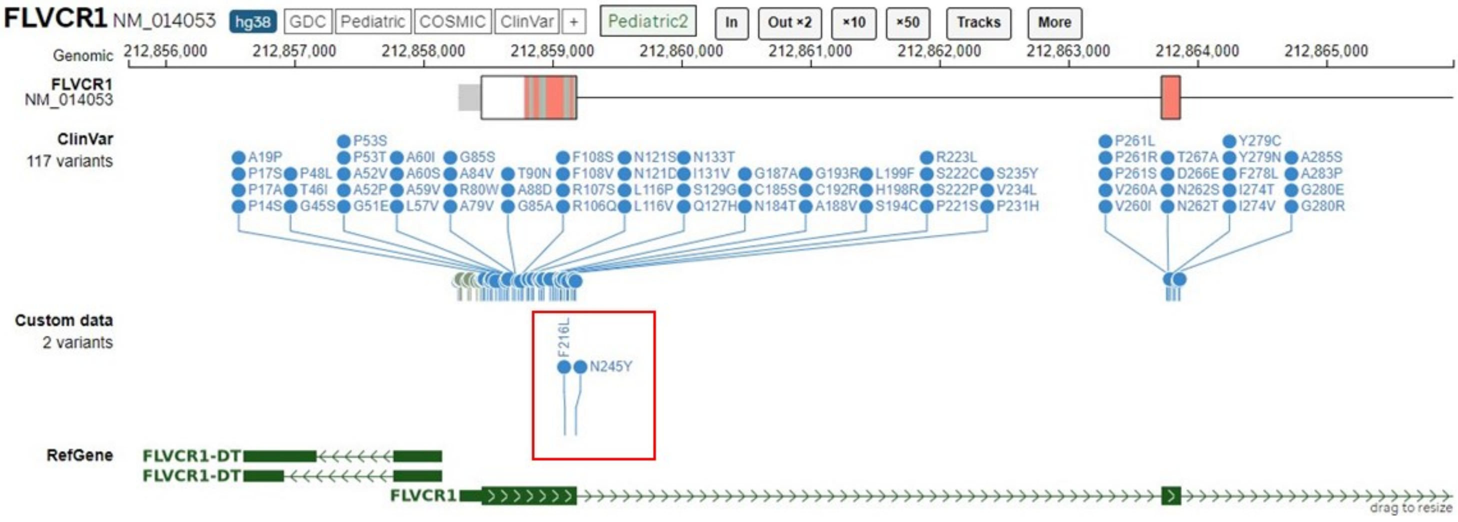

C.

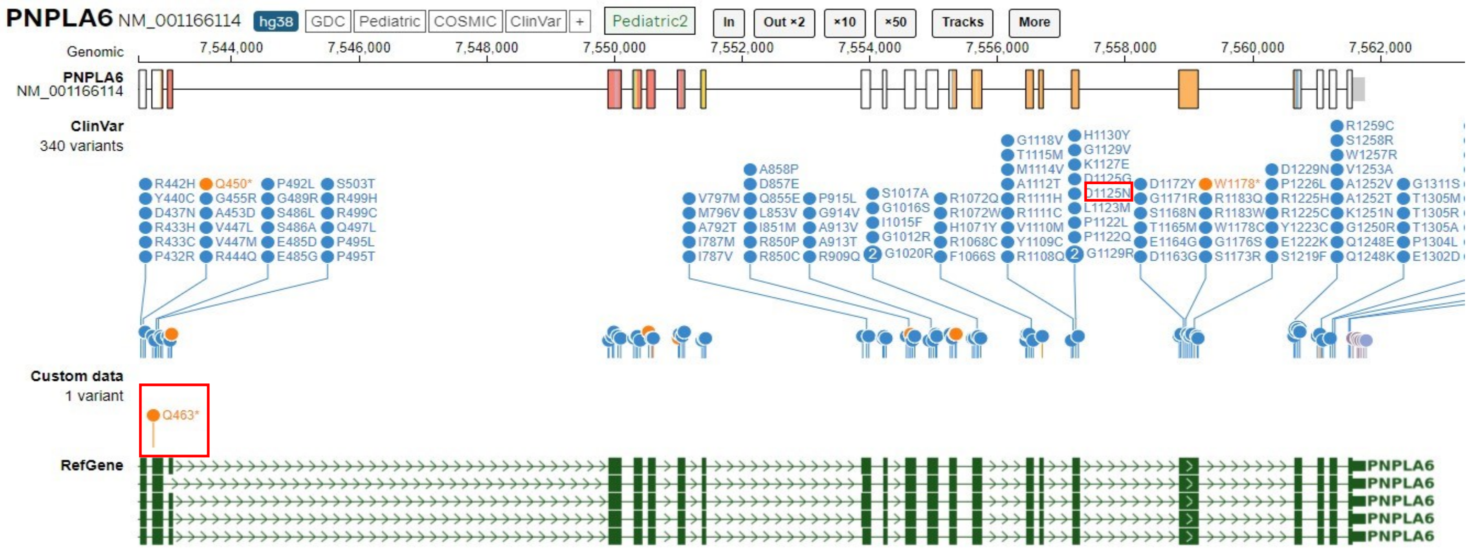

Figure S4. The figure was created using the Proteinpaint website (https://viz.stjude.cloud/tools/proteinpaint). The diagrams show sequence variants in *ABHD12* (A), *FLVCR1* (B), and *PNPLA6* (C) genes located across the genomic

sequences of the respective genes. Yellow corresponds to nonsense variants, and blue to missense variants. Variants identified in patients described in the manuscript are marked with a red box.
